# Effective cataract surgical and refractive error coverages in the state of Qatar

**DOI:** 10.1186/s12886-025-03915-1

**Published:** 2025-02-25

**Authors:** Shadi M AlAshwal, Muhammad Rabiu, Ian McCormick, Omar AlQahtani, Samya AlAbdulla, Halla Algadi, Suresh Kokku, Mohammed Hamad Al-Thani

**Affiliations:** 1 Ministry of Public Health, Doha, QA Qatar; 2 Noor Dubai Foundation, Dubai, AE United Arab Emirates; 3 London School of Hygiene & Tropical Medicine, International Centre for Eye Health, London, UK; 4 Hamad Medical Corporation, Doha, QA Qatar; 5 Primary Health Care Corporation, Doha, QA Qatar

**Keywords:** Cataract surgery, Effective coverage, Eye Health, Qatar, Refractive error, Visual acuity

## Abstract

**Purpose:**

To evaluate the progress in Qatar’s eye care since 2009, focusing on effective cataract surgical and refractive error coverages, leading to enhanced eye health strategies and action plans.

**Methods:**

A modified Rapid Assessment of Avoidable Blindness (RAAB) survey was employed using multi-stage sampling in all persons 50 years and older in Qatar. The study focused on uncorrected refractive errors, cataract surgery coverage and effectiveness, and visual acuity assessment.

**Results:**

There were 339 individuals out of 3,206 examined participants who underwent cataract surgery, out of which 66.1% of 559 operated eyes obtained good post-operative outcomes (presenting visual acuity ≥ 6/12). Age -sex - adjusted eCSC for a cataract surgical threshold < 6/12 was 61.2% (95%CI 54.9–67.4). A poor post-operative outcome (presenting visual acuity < 6/60) was observed in 9.3% of all operated eyes, lower than the 14.9% reported in 2009. Cataract surgical coverage at the 6/18 threshold showed good coverage (94%) improving since 2009 (87%). Effective refractive coverage (eREC) was 74.3% (95%CI 70.9–77.7). Effective coverage of both services was lower among Qatari women compared to other population groups.

**Conclusion:**

Qatar’s CSC improved since the 2009 RAAB, but there are disparities in effective coverage based on gender and nationality. WHO set a global target to achieve a 30%-point increase in eCSC and a 40%-point increase in eREC by 2030; accordingly, Qatar’s targets should be 91.2% and 100% retrospectively by 2030. To meet these targets, efforts are needed to improve the quality of cataract surgery and access to refractive correction.

## Introduction

In 2020, an estimated 15.2 million people worldwide were blind due to cataracts and 2.3 million caused by uncorrected refractive errors (URE) [1]. Over 1 billion people worldwide have vision impairment that could be prevented or remains untreated, mostly related to URE and untreated cataract contributing to 123.7 million and 65.2 million, respectively [2, 3]. 

In 2020 [4], the World Health Organization (WHO) in its 73rd World Health Assembly urged member countries to implement the recommendations in the World report on vision [2], which included the implementation of periodic population surveys to estimate VI and reporting effective coverage of cataract surgery and refractive error [4]. Subsequently, in 2021, member states at the 74th WHA endorsed the use of effective cataract surgical coverage (eCSC) and effective refractive error coverage (eREC) as indicators to measure countries’ progress in eye care as part of universal health coverage [4, 5]. Furthermore, in 2022, the United Nations identified these indicators as candidate indicators to measure progress towards the 2030 United Nations Sustainable Development Goals (SDGs) [5, 6]. Healthcare governance in Qatar is exercised through the Ministry of Public Health (MOPH), it supervises the country’s two principal health care providers: Hamad Medical Corporation (HMC) and Primary Health Care Corporation (PHCC) [7]. Eye care services are mostly provided via HMC which is the public sector provider of cataract operations and ophthalmic sub-specialty services; in addition, there are a few private hospitals that offer cataract surgery [8, 9]. The Rapid Assessment of Avoidable Blindness (RAAB) is a population based cross-sectional survey with a standardized study design, examination procedures, mobile data collection and analysis [10]. 

In 2009, Qatar conducted a RAAB study, revealing a blindness prevalence of 1.28% among people aged 50 years and older. URE and cataract caused 42% and 33% of VI, respectively. Cataract surgical coverage (CSC) at the < 6/18 surgical threshold was 86.6% (unpublished report). To track the country’s eye care progress since the last RAAB study, including on cataract service coverage, the MOPH and PHCC conducted a follow-up survey.

## Materials and methods

A modified RAAB survey was conducted across the state of Qatar from May to September 2022 and March to June 2023 (allowing for a pause in data collection October 2022 to February 2023 during the period of the FIFA World Cup). A minimum sample size of 5060 was calculated using the RAAB sample size calculator, based on a 1.28% blindness prevalence from the 2009 RAAB study, with the aim of achieving 0.38% precision and a 95% confidence level, factoring in a design effect of 1.4 and 10% non-response rate. Participants were selected using a stratified two-stage cluster random sampling. At the first stage, communities were used as primary sampling units (PSUs) and stratified into Qatari, and non-Qatari. The selection was divided proportionally between Qataris and non-Qataris in a 1:2.2 ratio; thus, out of 145 PSUs required for the study, 100 PSUs were assigned to the predominantly non-Qatari communities and 45 PSUs to the predominately Qatari communities. For each stratum, the number of PSUs was randomly selected from the strata’s PSUs with probability proportional to population size (PPS). The second stage was different from a typical RAAB sampling approach. The Qatari Planning and Statistics Authority randomly selected 35 eligible persons per chosen PSU from an individual-level list of residents [11]. The following Individuals were excluded (1) less than 50 years old, (2) resided in Qatar for less than six months, (visitors/ short contact workers), or (3) had COVID-19 or recent contact with persons who had COVID-19 confirmed infection. A certified trainer trained 10 teams (each with an ophthalmologist and 2 nurses) on survey design, data entry, and examination protocols. The training done in two batches aimed for Kappa coefficients above 0.60 for Inter-Observer Variation accuracy for VA, lens assessment, and causes of vision loss. Each selected survey participant was contacted by phone to seek his/her consent and then booked to attend one of 10 health centers selected for the survey. Upon arrival at the health center, participants confirmed their identity and consented to participate in the study in writing. Invited participants that could not come to the health center were offered a visit at home to be examined. Using the RAAB7 Android application on tablets, data was entered, including examination results and medical history.

All participants underwent VA measurement (uncorrected, corrected, pinhole) deploying the Peek Vision acuity test in the RAAB7 application. Lens was assessed by an ophthalmologist to determine the presence of aphakia, pseudophakia, or lens opacity. Any participant with a presenting VA (PVA) < 6/12 in an eye was further assessed by an ophthalmologist to determine the cause of poor vision, with pupil dilatation where needed. The RAAB methodology followed a WHO algorithm for determining the main cause(s) of vision impairment for eyes and person [12]. 

All Vision impairment was defined as combination of all levels of vision impairment excluding blindness, i.e., as a combination of mild, moderate, and severe VI. Mild VI = PVA < 6/12 − 6/18, Moderate VI = PVA < 6/18 − 6/60 and Severe VI = PVA < 6/60 − 3/60 in the better eye. Blindness was defined as a PVA is less than 3/60 in the better eye [12]. Cataract surgical coverage (CSC) and eCSC are indicators to capture the extent of cataract surgery reach [13]. 

**CSC** = (X + Y) / (X + Y + Z).

**eCSC** = (A + B) / (X + Y + Z).

**A** = Individuals with unilateral cataract surgery achieving post-operative VA ≥ 6/12 in the operated eye with pinhole VA < 6/12 in the other eye.

**B** = Individuals with bilateral cataract surgery achieving post-operative VA ≥ 6/12 in at least one eye.

**X** = Individuals with unilateral cataract surgery + pinhole VA < 6/12 in the other eye.

**Y** = Individuals with bilateral cataract surgery (regardless of VA).

**Z** = Individuals with pinhole VA < 6/12 in both eyes as cataract is the primary cause of visual impairment.

The relative quality gap (difference between eCSC and CSC) was calculated as (CSC–eCSC)/CSC, with lower values reflecting better quality of cataract surgical services. Post-operative cataract surgical outcomes were defined as good (PVA ≥ 6/12), borderline (PVA 6/18 to 6/60) or poor (PVA < 6/60) according to the WHO VA thresholds for each category [14]. Other indicators were Refractive error coverage (REC) and eREC, the latter measures the proportion of individuals who need and receive the refraction services, improving their uncorrected VA (UCVA) from worse than 6/12 to 6/12 or better. URE was defined as UCVA of < 6/12 in the better eye that could be improved to equal to or better than 6/12 by refraction (corrected VA [CVA]), or by placing a pinhole occluder in front of an eye (pinhole VA [PinVA]) [15–17]. 

REC = (A + B) / (A + B + C)

eREC = A / (A + B + C).

- **A** = Individuals with spectacles, or contact lenses, whose UCVA = < 6/12 in the better eye achieving CVA = 6/12 in the better eye (Met Need).
- **B** = Individuals with spectacles, or contact lenses, whose UCVA and CVA are < 6/12 in the better eye but improved to 6/12 or better with Pinhole (Undermet Need).
- **C** = Individuals with no spectacles, nor contact lenses, whose UCVA < 6/12 in the better eye, and PinVA is 6/12 or better in the better eye (Unmet Need).

The study obtained ethical approval from the PHCC. Data were recorded on encrypted, password-protected mobile data collection devices followed by a secure upload directly to an encrypted server. Access to the data was limited to the Principal Investigator (PI), RAAB trainer, and selected technical staff. After the survey, identifiable data was removed.

## Results

**Fig. 1:**
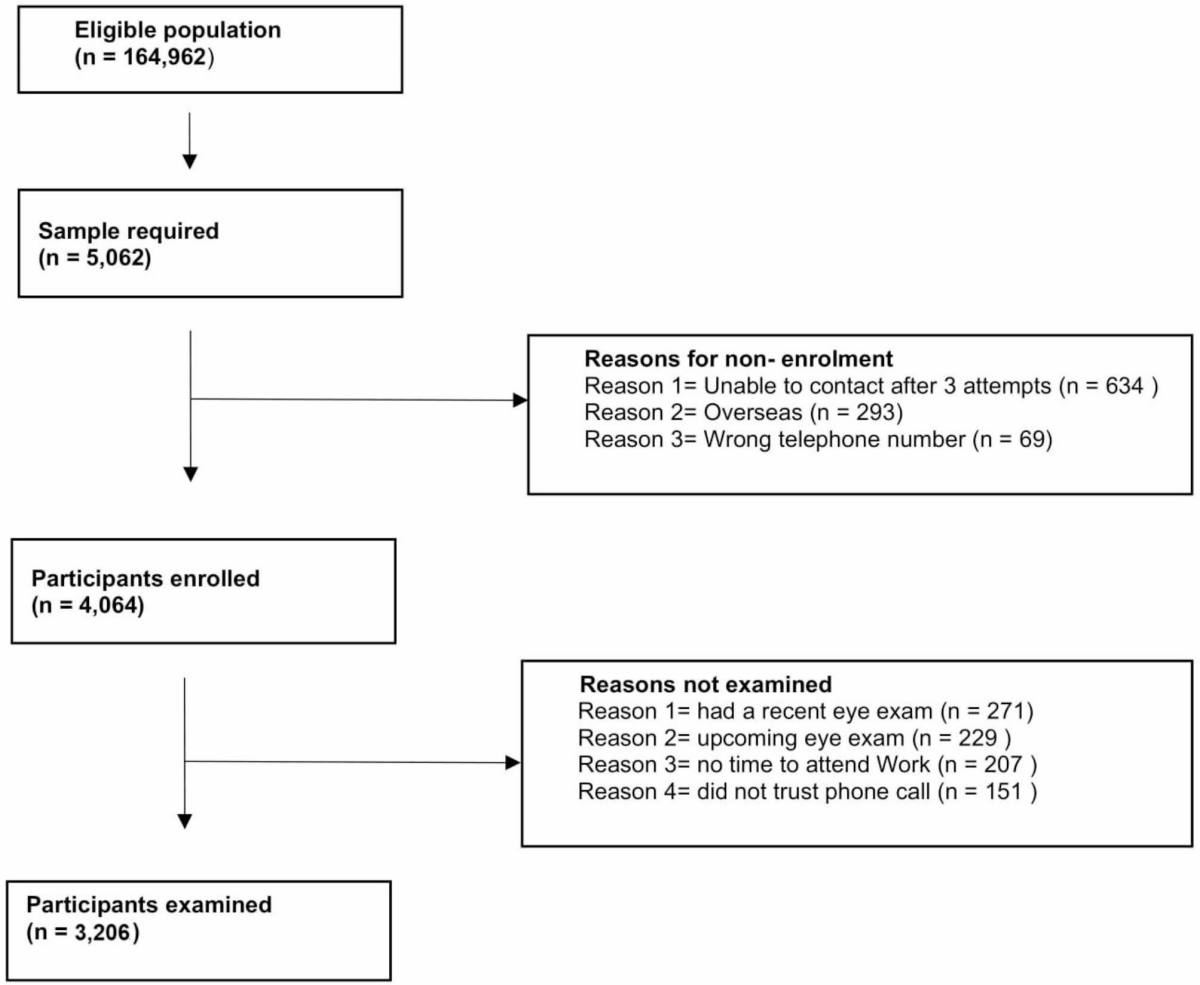
Flow diagram describing number of participants enrolled and examined and reasons for non-enrolment and non-examination

**Fig. 2:**
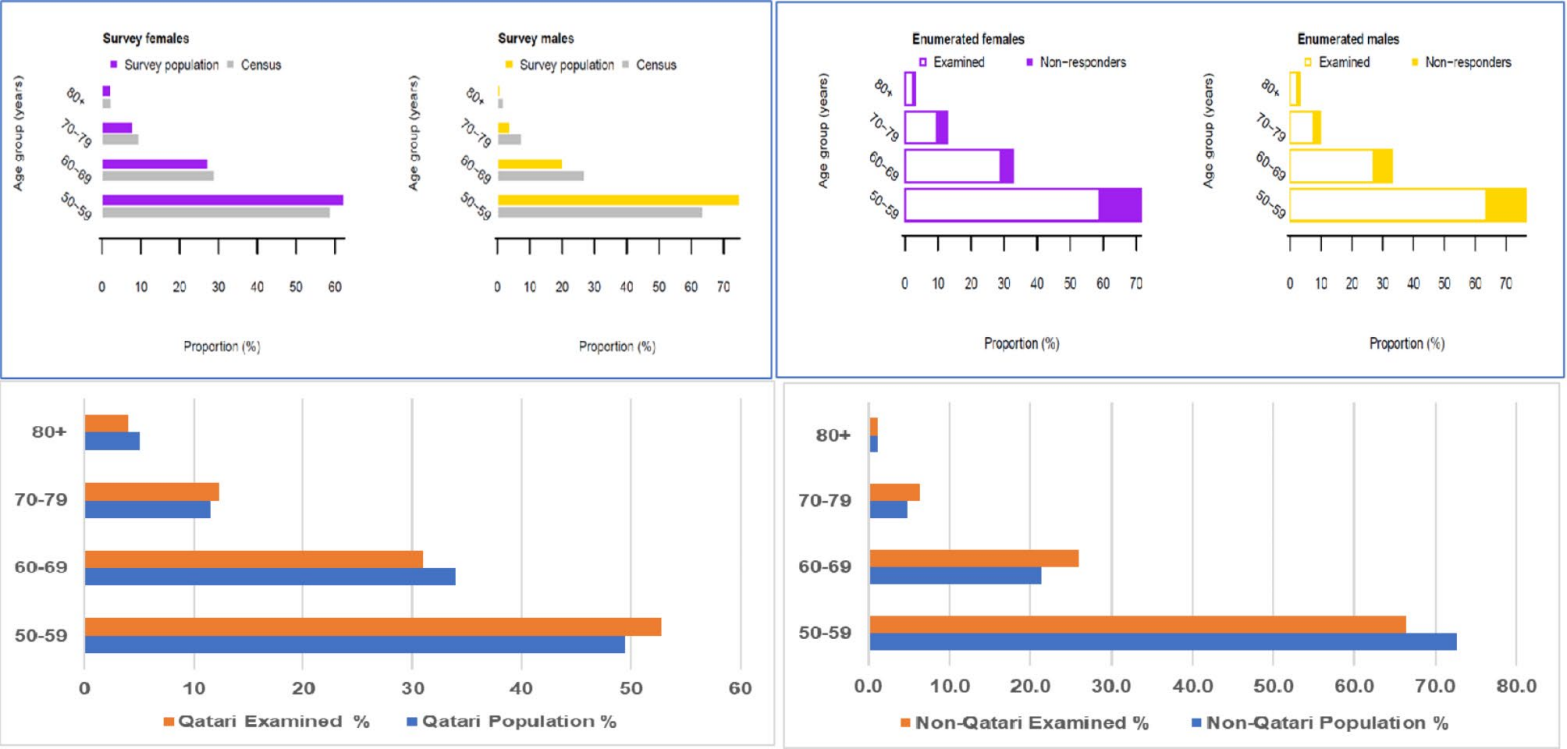
Top left shows the age-sex pyramid of males and females for the total sample population, top right shows bar charts of examined participants among the enrolled study participants for males and females, downright shows the percentage of population and examined by age group for the Qatari Stratum. Last part on the down left part, it shows how much the percentage of population and examined participants by the age group for the Non-Qatari Stratum

Out of the target sample size of 5060, 4064 (80.3%) participants were enrolled in the survey and, among those enrolled, 3206 people were examined, (59% were males and 35% were Qataris), a response rate of 78.9%. Figure 1 shows the reasons for non-enrolment and non-examination. The proportion of males and females in each 10-year age group examined was similar to the corresponding age-sex proportions in the total population (Fig. 2). The prevalence of bilateral blindness was 0.4% (95%CI 0.2–0.7), severe VI was 0.3% (95%CI 0.1–0.5), moderate VI was 3.8% (95%CI 3.0-4.5), and mild VI was 5.2% (95%CI 4.3–6.1). The main causes of all VI are described in Table 1. The causes of blindness and VI will be addressed in a related publication.

**Table 1:** Percentage of main causes of all vision impairment (presenting visual acuity worse than 6/12 and better than or equal to 3/60) among survey sample by nationality

Causes	Qatari		Non-Qatari		Total	
	Number	%	Number	%	Number	%
Uncorrected refractive error	89	47.3	96	70.6	185	57.1
Cataract	38	20.2	19	14.0	57	17.6
Diabetic retinopathy	17	9.0	5	3.7	22	6.8
Other posterior segment diseases	8	4.3	6	4.4	14	4.3
Corneal opacity	8	4.3	4	2.9	12	3.7
Globe/ Brain diseases	9	4.8	2	1.5	11	3.4
Age related macular degeneration	8	4.3	3	2.2	11	3.4
Cataract surgery complications	5	2.7	1	0.7	6	1.9
Glaucoma	4	2.1	0	0.0	4	1.2
Trachoma	1	0.5	0	0.0	1	0.3
Phthisis	1	0.5	0	0.0	1	0.3
Total	188	100.0	136	100.0	324	100.0

### Cataract

The unmet need for cataract surgery among people aged 50 years and older in Qatar included an estimated 1,847 people who required bilateral surgery for all levels of cataract-related vision impairment < 6/12, with a further 3,879 surgeries required for unilateral cataract < 6/12 (Table 2).In the sample, there were 561 operated eyes in 339 individuals who had received cataract surgery at some time prior to the survey. A post-operative good outcome was achieved in 66.1% of all operated eyes, with variation by nationality (non-Qataris 73.9% vs. Qataris 58.3% good outcomes) and sex (males 70.5% vs. females 60.9% good outcomes). A poor post-operative outcome was observed in 9.3% of all operated eyes (11.9% in Qataris vs. 6.7% in non-Qataris) (Table 3), which is lower than the 14.9% reported in the 2009 survey sample of 390 operated eyes [18]. The causes of poor post-operative vision outcomes were ocular comorbidity (50.9%), surgical complications (31.5%), and URE (17.6%).

**Table 2:** Age-sex adjusted prevalence and magnitude of bilateral cataract, by nationality and sex

Cataract surgical threshold (Pinhole VA)	Qataris				Non-Qataris				Total				
	Males %	Females %	Total %	Extrapolated Magnitude	Males %	Females %	Total %	Extrapolated Magnitude	Males %	Females %	Total %	Extrapolated Magnitude	
< 6/60	0.0	0.2	0.1	44	0.0	0.0	0.0	30	0.0	0.1	0.1	98	
< 6/18	0.9	0.6	0.8	388	0.1	0.4	0.2	259	0.3	0.5	0.4	597	
< 6/12	2.3	2.7	2.4	1145	0.5	0.9	0.6	679	1.0	1.6	1.1	1847	

**Table 3:** Post-operative presenting visual acuity among cataract operated participants in the Survey Sample, by Nationality and Sex Population subgroups

Presenting VA outcome	Qataris			Non-Qataris			Total		
	Males %	Females %	Total %	Males %	Females %	Total %	Males %	Females %	Total %
Good (6/12)	75 (62)	87 (55.4)	162 (58.3)	142 (75.4)	67 (69.8)	209 (73.9)	217 (70.5)	154 (60.9)	371 (66.1)
Borderline (< 6/12 − 6/60)	32 (26.4)	51 (32.5)	83 (29.9)	34 (18.2)	21 (21.9)	55 (19.4)	66 (21.4)	72 (28.5)	138 (24.6)
Poor (< 6/60)	14 (11.6)	19 (12.1)	33 (11.9)	11 (5.9)	8 (8.3)	19 (6.7)	25 (8.1)	27 (10.7)	52 (9.3)
Total	121	157	278	187	96	283	308	253	561

The age and sex-adjusted CSC for a cataract surgical threshold < 6/12 was 85.6% (95% CI 81.1–90.0), while the age and sex-adjusted eCSC for a cataract surgical threshold < 6/12 was 61.2% (95%CI 54.9–67.4). The eCSC for a cataract surgical threshold < 6/12 was higher in non-Qataris (72.4% [95% CI 64.8–79.9]) than Qataris (52.0% [95% CI 42.8–61.2]) (Tables 4 and 5). Overall, the relative quality gap at this threshold was 28.5% but this was worse for Qataris (37.8%) and, better for non-Qataris (18.6%). The proportions of surgeries done in government clinics were similar for Qataris (54%) and non-Qataris (60%) (Table 4).

**Table 4:** Description of Cataract operated participants and age-sex adjusted Cataract Surgical Coverage (CSC) and effective Cataract Surgical Coverage (eCSC) at the < 6/12 Cataract Surgical threshold, by Nationality and Sex Population subgroups

Variable	Qataris %			Non-Qataris %			Total %		
	Males	Females	Total	Males	Females	Total	Males	Females	Total
Median age sample (IQR)	58 (53–66)	60 (54–68)	59 (54–67)	56 (52–62)	55 (51–61)	56 (52–62)	57 (53–63)	57 (53-64.75)	57 (53–64)
Number of operated people	71	98	169	113	57	170	184	155	339
Number of operated eyes	119	157	278	187	96	283	306	253	559
Median age cataract operated (one or both eyes) (IQR)	68 (62.5–77)	70.5 (65–77)	70 (64–77)	67 (59–73)	68 (62–74)	68 (60-73.75)	67 (61–75)	70 (64.5–77)	68 (63–76)
Median time since surgery in years (IQR)	6 (3–10)	4 (2–10)	5 (2–10)	4 (2-9.5)	4 (2.75-8)	4 (2–9)	5 (2–10)	4 (2–10)	5 (2–10)
% of Governmental hospital	56	52	54	64	54	60	61	53	57
Cataract surgical coverage < 6/12	85.5 (76.7–94.4)	80.6 (71.5–897.)	83.5 (76.5–90.6)	89.9 (83.9–95.8)	87.9 (79.3–96.5)	88.9 (83.8–94.0)	87.8 (82.7–92.8)	83.2 (76.6–89.8)	85.6 (81.1–90.0)
Effective cataract surgical coverage < 6/12	55.5 (41.5–69.4)	46.8 (37.1–56.6)	52.0 (42.8–61.2)	78.0 (69.8–86.3)	66.9 (54.0-79.9)	72.4 (64.8–79.9)	67.5 (59.8–75.2)	54.3 (46.4–62.2)	61.2 (54.9–67.4)
Relative Quality Gap	35.1	41.9	37.8	13.2	23.9	18.6	23.1	34.7	28.5
Good outcome (%)	62.0	55.4	58.3	75.9	69.8	73.9	70.5	60.9	66.1

**Table 5:** Age-sex adjusted Cataract Surgical Coverage and Effective Cataract Surgical Coverage at the person level, by Nationality and Sex Population subgroups

CSC/eCSC	Nationality		Sex		Total% (95%CI)	
	Qatari % (95%CI)	Non-Qatari % (95%CI)	Males% (95%CI)	Females % (95%CI)		
Cataract surgical threshold Pinhole VA < 6/12						
CSC	83.5 (76.5–90.6)	88.9 (83.8–94.0)	87.8 (82.7–92.8)	83.2 (76.6–89.8)	85.6 (81.1–90)	
eCSC	52.0 (42.8–61.2)	72.4 (64.8–79.9)	67.5 (59.8–75.2)	54.3 (46.3–62.9)	61.2 (54.9–67.4)	
Relative quality gap (%)	37.7	18.6	23.1	34.7	28.5	
Cataract surgical threshold Pinhole VA < 6/18						
CSC	93.3 (88.9–97.8)	95.4 (92.2–98.7)	95.4 (91.6–99.2)	93.3 (88.9–97.6)	94.4 (91.7–97.2)	
eCSC	58.5 (48.7–68.3)	78.6 (71.6–85.5)	72.7 (65.1–80.4)	63.0 (54.7–71.3)	68.3 (62.0–74.5)	
Relative quality gap (%)	37.2	17.6	23.7	32,5	27.7	
Cataract surgical threshold Pinhole VA < 6/60						
CSC	98.5 (96.2–100)	99.5 (97.9–100.0)	99.3 (97.9–100.0)	97.8 (95.3–100)	98.6 (97.3–100)	
eCSC	62.3 (52.3–72.7)	83.1 (76.8–89.5)	76.3 (69.1–83.4)	67.6 (59.2–76.1)	72.4 (66.2–78.5)	
Relative quality gap (%)	36.7	16.4	23.2	30.9	26.6	

* CSC = cataract surgical coverage, eCSC = effective cataract surgical coverage

### Refractive error

Over a third of survey participants used distance glasses (36.8%), and over two-thirds used reading glasses (69.6%). Among the population 50 years and older, 77.4% (95% CI 66.6–88.2) had no need for distance refractive correction according to the definition of eREC set out above. Among the remainder, eREC for distance vision was 74.3% (95%CI 70.9–77.7). Comparing population subgroups by nationality and sex, Qatari females had lower distance eREC than all other groups and Qatari males had lower distance eREC than non-Qatari males (Table 6). The unmet need for distance refractive error correction was estimated at 8,290 people, (5.0%; 95%CI 2.7-7.4%), with a further 1,286 people (0.8%; 95%CI 0.0-3.8%) with sub-optimal correction (undermet need) (Table 6). Given the absence of 6/12 data, in the 2009 RAAB study, comparison of refractive error estimates with the 2009 survey is not possible; distance spectacle use among the sample (irrespective of need) increased from 20.6% in 2009 to 36.8%.

**Table 6:** Age-Sex Adjusted Refractive Error Coverage and Effective Refractive Error Coverage among people aged 50 years and older by nationality and sex

REC and eREC*	Qataris %			Non-Qataris %			Total %			
	Males (95%CI)	Females (95%CI)	Total (95%CI)	Males (95%CI)	Females (95%CI)	Total (95%CI)	Males (95%CI)	Females (95%CI)		Total (95%CI)
REC	72.2 (49.4–68.5)	58.9 (49.4–68.5)	66.6 (60.7–72.5)	85.7 (81.7–89.7)	82.6 (77.5–87.7)	84.2 (81.0-87.5)	81.6 (78.0-85.1)	73.0 (67.6–78.5)	77.8 (74.5–81.0)	
eREC	67.5 (60.2–74.7)	55.3 (46.7–64.7)	62.3 (56.3–68.3)	83.0 (78.7–87.3)	78.8 (73.3–87.3)	81.0 (77.6–84.5)	78.3 (74.4–82.1)	69.4 (63.8–75.0)	74.3 (70.9–77.7)	
RQG	6.5	6.1	6.4	3.2	4.6	3.8	4.0	4.9	4.4	

* REC: Refractive error coverage; eREC: effective Refractive error coverage, RQG: Relative Quality Gap

## Discussion

This survey found a low prevalence of blindness and vision impairment in the population aged 50 years and older in Qatar, with over 90% having a distance PVA of 6/12 or better. According to 2020 census data, there is a difference in the age profile of the two main population subgroups, namely Qatari nationals and non-Qataris [11]. Among the population 50 years and older, we found the relatively younger non-Qatari population had a higher proportion of VI due to URE, whereas the older Qatari population had a higher proportion of VI due cataract (Qatari population 21% compared to 13% in non-Qataris). CSC < 6/12 was consistent across population subgroups at around 85% but differed in eCSC. Good outcomes were more common among operated eyes in the non-Qatari sample than the Qatari sample and, accordingly, eCSC was significantly lower in the Qatari population than the non-Qatari population. While the Qatari sample was older than the non-Qatari (reflecting the underlying demographic profile of Qatar), the ages of those operated in each group, and the average time since surgery, were similar. Qatari females were on average the oldest group among those operated but their average time since surgery was similar to groups with better outcomes (Table 4). The 2009 RAAB survey did not access VA at the 6/12 threshold, therefore the eCSC cannot be reported in 2009. However, point estimates of CSC at the 6/18 cataract surgical threshold showed good coverage improving further since 2009 (87–94%) [18]. In 2009, HMC performed 776 operations. By 2023, this number had increased to 2,638 including 2,139 for non-Qataris and 499 for Qataris. (unpublished data, HMC).

WHO set a global target to achieve a 30%-point increase in eCSC by 2030 and noted that increases should be equitable across population subgroups. Accordingly, Qatar’s eCSC target should be 91.2% by 2030 [4]. Given the already uniformly high level of crude coverage (85.6%), almost all of this increase could be achieved by eliminating the relative quality gap, with an emphasis on improving outcomes in Qatari nationals in particular.

A comparison of the proportion of post-operative good outcomes in 2023 and 2009 was not possible; however, the decrease in poor post-operative outcomes between 2009 and 2023 (14.9–9.3%) indicates that quality may be improving. Given the similar age profiles and time since surgery of operated participants by nationality, further research is needed to understand the reasons behind worse post-operative outcomes in the Qatari population, including active monitoring of health facility data for results of recent surgeries, as is done in, e.g., the United Kingdom’s National Ophthalmology Database (NOD) cataract audit system [19]. Distance eREC was 74.3% (95% CI 70.9–77.7), taking into consideration that the estimate only relates to people aged 50 years and older.That was higher than 2021 estimate of global eREC (42.9% [38.0-47.8]) in the same age group and similar to the 79.1% (95% CI [72.4–85.0]) estimate for the High-Income Global Burden of Disease (GBD) region [17]. eREC should reach 100% to align with the 2030 global target for the indicator [4]. Strategies to increase eREC need to take into consideration the demographics population groups that demonstrated lower effective coverage, i.e. Qataris and women, and how these two characteristics intersect eREC was almost 30% points lower among Qatari females (55.3%) compared to non-Qatari males (83.0%).

Although the response rate of the study was less than optimal, the sample was representative of the sex and nationality profile of the population of interest as the proportions of males and Qatari nationals examined reflected the proportions for the same age group in the population. The less-than-optimal response rate was related to the participant recruitment strategy, i.e., inviting them to their health centers instead of using the typical door-door approach in RAAB studies. 20% of the selected sample were unreachable by telephone, and 20% declined participation, citing reasons such as disinterest or prior/ future appointments with their ophthalmologist. These recruitment challenges are more pronounced in high-income countries with excellent access to eye health care services. The survey was designed to reflect the census ratio of Qatari to non-Qatari populations at the first stage of sampling, however, the sample size was not calculated to allow for subgroup comparisons or defined precision of estimates in subgroups facilities outside of Qatar, but future surveys should confirm this.

In conclusion, the RAAB survey highlighted an improvement of CSC since 2009 and eREC comparable with a modeled estimate for the GBD High-Income super region. However, disaggregated eCSC and eREC estimates revealed that women and Qatari nationals had reduced access to quality cataract surgery and refraction services. Thus, the health authorities have to invest more in setting strategies that minimize these gaps. This may include improving access to care and early detection and treatment of other chronic/age-related eye diseases like glaucoma, diabetic retinopathy, etc. To fully leverage the survey results, an Eye Care Situational Analysis Tool (ECSAT) exercise is advised, paving the way for tailored recommendations to improve eye care in Qatar [14, 20]. 

## Data Availability

Data supporting the findings of this study are available from the corresponding author upon reasonable request.
